# Supplementary material for: Comprehensive Annotation and Functional Exploration of MicroRNAs in Lettuce
Source: Front Plant Sci. 2021 Dec 24;12:781836. doi: 10.3389/fpls.2021.781836 (PMC8739914; doi:10.3389/fpls.2021.781836)
Supplement: Supplementary file 3 [file Data_Sheet_9.ZIP › GSM2230753.plot/Lsa-miR403_Lsat_1_v5_gn_4_22000.1_3565_TPlot.pdf]

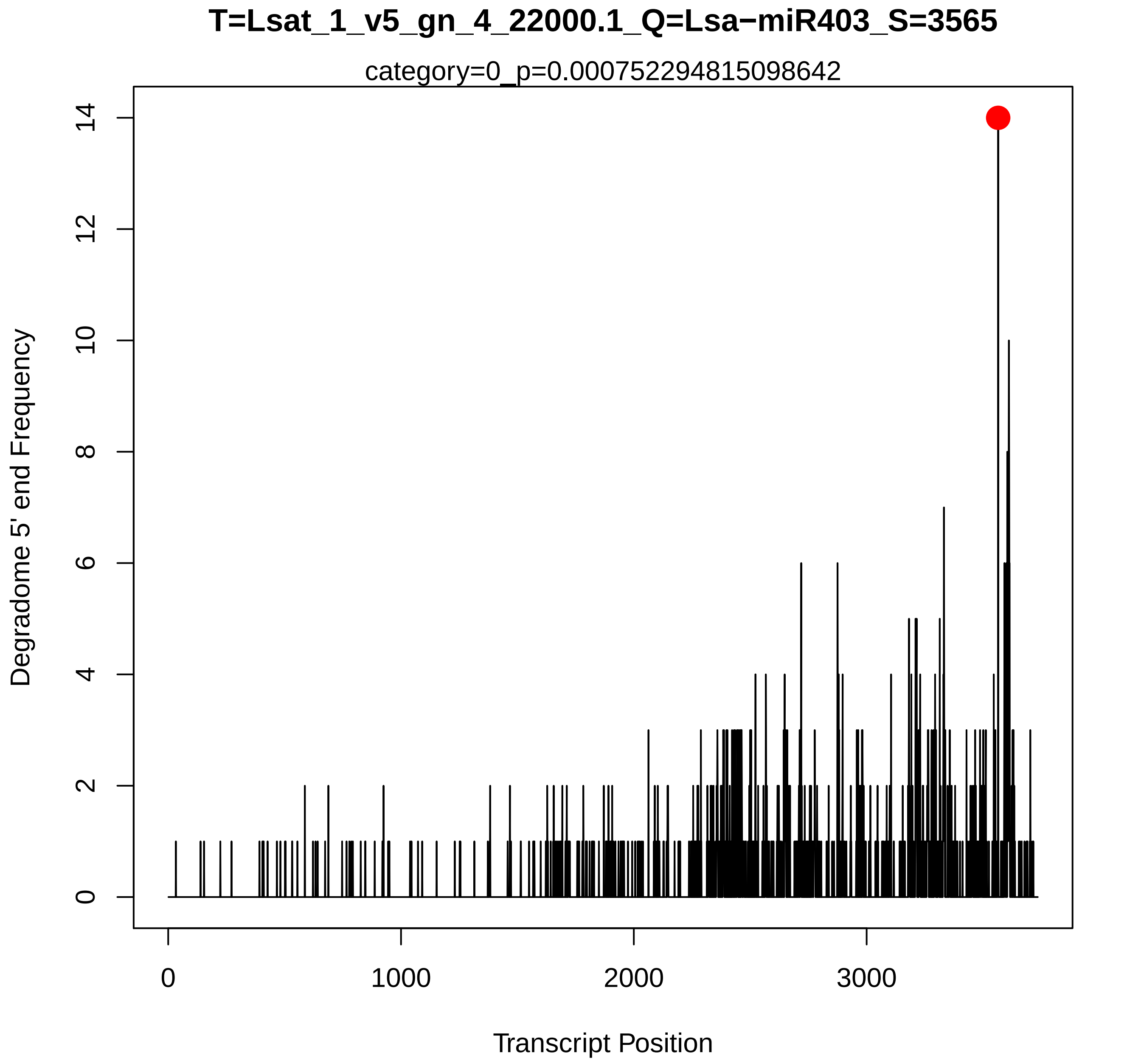
T=Lsat_1_v5_gn_4_22000.1_Q=Lsa−miR403_S=3565
category=0_p=0.000752294815098642
Degradome 5' end Frequency
0
2
4
6
8
10
12
14
0
1000
2000
3000
Transcript Position
